# Linear versus Circular Stapler for Gastrojejunal Anastomosis in Laparoscopic Roux-En-Y Gastric Bypass: An Analysis of 211 Cases

**DOI:** 10.1155/2020/4090797

**Published:** 2020-07-30

**Authors:** Laurin Burla, Pascal Weibel, Cornelia Baum, Markus Huber, Thomas Gürtler, Markus Weber

**Affiliations:** Department of Visceral, Thoracic and Vascular Surgery, Triemli Hospital, Birmensdorferstrasse 497, Zurich 8063, Switzerland

## Abstract

**Purpose:**

Although laparoscopic Roux-en-Y gastric bypass (LRYGB) is a frequently performed bariatric procedure, there is still no consensus on its technical implementation.

**Methods:**

211 patients treated with LRYGB in a single institution between March 2011 and October 2016 were analyzed retrospectively. A subgroup analysis for the linear (LSA) versus circular stapler technique (CSA) for gastrojejunal anastomosis (GJA) was performed to evaluate complications and outcomes.

**Results:**

128 (60.6%) patients received GJA with CSA and 83 (39.4%) with LSA. Average weight loss one year after surgery, respectively, BMI after one year of follow-up (kg/m^2^), showed no significant difference. Median surgery time was significantly shorter in the LSA group. If the procedure was performed with CSA, significantly more wound infections occurred.

**Conclusions:**

Both the circular and the linear stapler techniques for gastrojejunal anastomosis in laparoscopic Roux-en-Y gastric bypass are safe methods with comparable outcomes. A disadvantage of CSA is the significantly higher rate of wound infections, a circumstance which requires increased attention.

## 1. Introduction

Obesity, defined as a BMI > 30 (kg/m^2^), is on the rise [1]; in Switzerland, the condition has increased since 1992 from 5.4% (men 6.1% and women 4.7%) to 11.3% (men 12.3% and women 10.2%) of the total population in 2017 [2].

As a risk factor for multiple physical and psychological obesity-related diseases, it is responsible for a high level of suffering. This leads not only to a high burden of disease for the affected patients and the health care system, but also to a considerable socioeconomic load. Bariatric surgery is a highly efficient method to treat obesity and its comorbidities sustainably [3, 4]. Although sleeve gastrectomy is the most common bariatric procedure worldwide, LRYGB is preferred in two-thirds of cases in Switzerland [5].

In terms of recent data, LRYGB appears to be superior to sleeve gastrectomy, particularly regarding diabetes mellitus and associated comorbidities [6–9].

**Figure 1:**
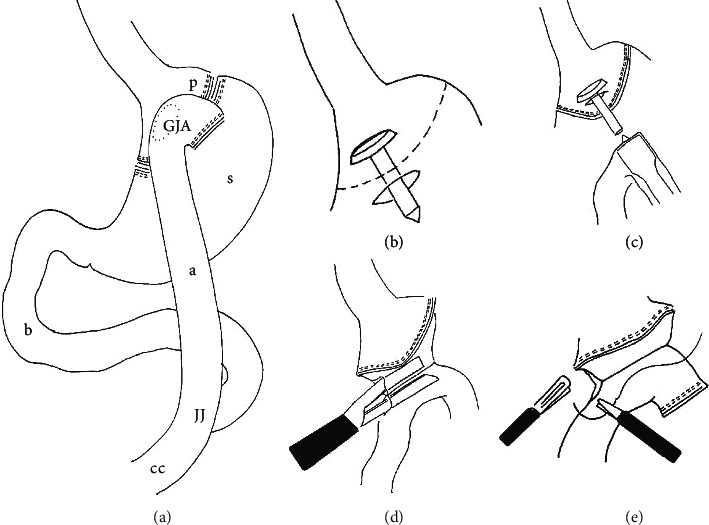
Circular and linear stapler techniques for gastrojejunal anastomosis. (a) Roux-en-Y gastric bypass. CSA technique: (b) inserting the stapler head in the stomach before gastric pouch formation; (c) forming the gastric pouch and leading out the central rod from the pouch, inserting the circular stapler into the jejunal loop, and performing anastomosis. LSA technique: (d) after forming the stomach pouch, the jejunal loop is brought near and fixed with a holding thread, opening of the stomach pouch and the jejunal loop and introduction of the linear stacker, and preparation of the anastomosis; (e) closure of the opening for the stacker. GJA: gastrojejunal anastomosis, p: stomach pouch, s: residual stomach, b: biliary limb, a: alimentary limb, jj: jejunojejunostomy, and cc: common channel (the anastomosis was shown here once in front of and once behind the stapler suture row of the stomach pouch for simpler illustration).

An essential step of this surgery is the gastrojejunal anastomosis (GJA). Nowadays, GJA is mostly performed either by circular (CSA) or linear stapler (LSA) than with a hand-sewn anastomosis (HSA) (Figure 1).

In 2014, our clinic switched to the linear stapler. The key factors for the change from circular to linear stapler were first study results, which indicated fewer wound infections and fewer stenoses in the GJA in favour of the linear stapler, and, subjectively, the linear stapler seemed to have an easier intraoperative handling [10, 11].

However, the choice of technique still depends on the surgeon's preferences and training. A standard has not yet been established [12].

Common early complications of LRYGB are wound infections, pulmonary embolism, and anastomotic insufficiency. The treatment of wound infections in obese patients can be challenging and are not always easy to treat. Late complications include ulcerations and internal hernias.

The aim of this study is to compare patients treated with LRYGB with regard to the stapler technique of GJA and its complications.

## 2. Materials and Methods

### 2.1. Population and Indication

229 patients who underwent LRYGB from March 2011 to October 2016 at a single institution were reviewed. Each patient had reached the age of 18. The indications as well as contraindications were based on the SMOB criteria (Table 1). The most important conditions for the indication include a BMI (body mass index) above 35 kg/m^2^ and a period of two years of unsuccessful adequate conservative therapy for weight reduction, respectively, one year at BMI ≥ 50 kg/m^2^. In addition, in this study, the exclusion was also given for a loss of follow-up or a revisional surgery (“redo”) intervention.

The preoperative examinations were carried out according to in-house instructions, which include a blood sample, an X-ray of the thorax, a sonography of the upper abdomen checking for gallstones, liver size, cirrhosis, or ascites, and a gastroscopy. An informative discussion was conducted by the nutritionist.

### 2.2. Surgical Technique

The patients received perioperative antibiotic prophylaxis with cefuroxime 3 g intravenously and thromboembolism prophylaxis with enoxaparin 60 mg subcutaneously. The target range for perioperative glucose control was 5–10 mmol/L and for temperature >35.5°C. The operation was conducted using standardized surgical steps. All interventions were performed by the same experienced surgeon in bariatric surgery.

The laparoscopic accesses were made in both groups via one 5 mm and four 12 mm trocars. The GJA was conducted either with a 25 mm circular stapler [13] or a 45 mm linear stapler [14]. Initially, the GJA was carried out with the circular stapler, and from February 2014 onwards, there was a consistent change to the linear stapler.

For the circular stapler, a minilaparotomy was performed to insert the stapler and, after the GJA has been made, to remove it. No cover was used for the stapler or the wound. The wound was rinsed with a saline solution, and topical antibiotics were not administered. The incision of the insertion/exit point of the circular stapler was left open at the end of surgery and covered with moist compresses. Irrigation of the wound and dressing changes were made once a day, and the wound was secondarily closed with wound strips at the day of discharge. All other wounds, whether the CSA or LSA subgroup, were primarily closed with skin clips.

### 2.3. Postoperative Care

All patients underwent the same postoperative care involving no placement of a nasogastric tube and early oral nutrition from the first postoperative day on. A PPI infusion pump was switched to pantoprazole 40 mg granules orally on the second postoperative day. Discharge was usually planned from the fourth postoperative day. Medication with pantoprazole was continued for 6 weeks and thromboembolism prophylaxis for 3 weeks. Postoperative follow-up after 6 weeks was performed during the surgeon's office hours. Further regular checkups were carried out by the referring endocrinologist and a nutritionist every 3 months in the first year and once a year for 5 postoperative years. Early complications were defined as such if they occurred within 30 days after surgery. A wound infection was diagnosed when local clinical signs of infection led to a change in the procedure: spreading of the wound and/or antibiotic therapy.

### 2.4. Data Collection and Statistical Analysis

The data were collected retrospectively from the clinic's own information system or then after discharge from the treating general practitioner or the nutritionist.

The statistical evaluation was carried out by an independent statistician using SPSS, version 20. For categorical data, a chi-square (if necessary with Yates' correction)/Fisher's exact test was used and for continuous data, a Mann–Whitney *U* test. A *p* value ≤0.05 was considered statistically significant. A subgroup analysis for the linear versus circular stapler technique for GJA was performed. The primary endpoint was the occurrence of a wound infection. Secondary endpoints were other early complications (anastomotic insufficiency and pulmonary embolism) and late complications (internal hernia or ulcerations), as well as time of surgery and achieved weight loss after one year of follow-up.

Before the start of the study, approval was granted by the responsible ethics committee and consent was obtained from the participants.

## 3. Results

Of 229 patients who qualified for LRYGB between March 2011 and October 2016, 211 patients were enrolled in this single-center retrospective study. 18 patients were excluded (8 patients with revisional surgery, 9 with too short follow-up time or loss of follow-up, and 1 patient with short-term refusal of surgery).

In 128 patients, CSA (60%) and in 83 patients LSA (40%) were performed.

Overall, there were no significant differences of data regarding demographics, disease, or main risk factors between the two groups. The median age in both groups was 41 years, and the percentage of women was 79% (CSA) vs. 76% (LSA). The preoperative BMI was 42.7 kg/m^2^ for CSA (interquartile range 39.0–47.3 kg/m^2^) and 41.8 kg/m^2^ for LSA (38.3–45.8 kg/m^2^), *p* value 0.4178. In the CSA subgroup, there was a nicotine abuse at 35.2% and a diabetes rate at 14.1% and, respectively, 37.3% (*p* value 0.8592) and 16.9% (*p* value 0.56) in the LSA subgroup (Table 2).

The median time of surgery was 141 minutes (CSA) vs. 119 minutes (LSA), *p* value <0.00001. In a subgroup analysis of the operation time with CSA with subdivision 2011/12 versus 2013/14, an average operation time of 155 versus 125 minutes was observed.

In the CSA group, 4 patients (3.1%) had to be converted to an open technique compared to none of the patients in the LSA, *p* value 0.1558.

Average weight loss one year after surgery (CSA 37 kg vs. LSA 35 kg, *p* value 0.0576), respectively, BMI (kg/m^2^) after one year of follow-up (CSA 28.6 kg/m^2^ vs. LSA 29.7 kg/m^2^, *p* value 0.2177), showed no significant difference. The mean follow-up was 47.0 months for CSA and 14.9 months for LSA (*p* value <0.00001) (Table 3).

Early complications were significantly increased in the CSA group, with 20 patients (15.6%) vs. 2 patients (2.4%) in the LSA group, *p* value 0.0022. This is mainly due to the significant difference in wound infections in favour of LSA (CSA 17 (13.3%) vs. LSA 2 (2.4%), *p* value 0.0143, approximate power (for 5% significance) of 76.05%). No significant differences in anastomosis insufficiencies (CSA (*n* = 2, 1.6%) vs. LSA (*n* = 0, 0%), *p* value 0.2525) or pulmonary embolism (CSA (*n* = 4, 1.6%) vs. LSA (*n* = 0, 0%), *p* value 0.1039) were found. In the CSA group, three patients suffered from the combination of two early complications (anastomotic insufficiency and pulmonary embolism, pulmonary embolism and wound infection, and anastomotic insufficiency and wound infection). There were no significant differences in late complications overall (CSA 15 (11.7%) vs. LSA 14 (16.9%), *p* value 0.3918) though gastric ulcers were significantly increased in LSA by 10 (12.0%) vs. CSA 4 (3.1%, *p* value 0.0238). Internal hernias occurred in CSA 12 (9.4%) vs. LSA 4 (4.8%, *p* value 0.3396) patients. In one case, both ulcer and internal hernia occurred in the CSA group (Table 4).

## 4. Discussion

The LRYGB is one of the most frequently used bariatric procedures worldwide. With a steady increase in morbid obesity and further improvements in the already demonstrable efficient surgical therapy, the number of interventions will continue to rise. An online survey (215 bariatric surgeons, 43% CSA, 41% LSA, and 21% hand-sewn anastomosis (HSA)), an analysis of the data from the Michigan Bariatric Surgery Collaborative (9904 patients, 66% CSA, 16% LSA, and 18% HSA), and a meta-analysis of 12 studies (13,626 patients (24% HSA, 50% CSA, and 26% LSA)) showed that the technique of GJA continues to vary widely [10, 15].

In this population, as in other studies, there was no relevant difference in the success of the LRYGB, whether CSA or LSA, and the weight loss was comparable. In addition, the significant reduction in operation time when using LSA (CSA 141 minutes vs. LSA 119 minutes) was also confirmed [11, 16–18]. The subgroup analysis with regard to the median operation time with CSA 2011/12 (155 minutes) versus 2013/14 (125 minutes) shows an improvement, but after almost 90 cases, it is still substantially above LSA.

As shown in other studies, there were no significant differences in anastomotic leakage [10, 11, 17].

What was noticed in most of the studies, however, was an increase in wound infections in circular stapler access, as this cannot be carried out by the trocar in comparison with the LSA and must be removed directly through the wound [10, 15, 17, 19]. There are studies which recommend preventive measures for wound infection prophylaxis.

Shabino et al. showed that stapler cover, wound irrigation, and wound antibiotic application in primary wound closure reduce the wound infection rate from 15% to 3.8% (*p* < 0.01) [20].

In comparison with this study, no stapler cover or local antibiotic application was used, but the wound was initially left open.

Vetter et al. showed a significant reduction of wound infections in secondary wound closure in stapler insertion sites with circular staplers (9.3% primary wound closure vs. 1% secondary wound closure, *p* value <0.001) [21]. In our population, despite secondary closure, the wound infection rate remained high (CSA 13.3% vs. LSA 2.4%, *p* value 0.0143).

The increased occurrence of gastric ulcers in LSA in our population is rather surprising, and we have no explanation for this result. No similar results were found in the literature. A meta-analysis showed an increased number of marginal ulcers overall in stapler anastomosis (CSA and LSA) vs. HSA, but there were no significant differences between CSA and LSA [22].

The limitations of this study lie, besides its retrospective study design, in a possible learning curve and also with regard to the operation time for the surgeon in favour of LSA, which definitively replaced CSA from January 2015.

The follow-up in the CSA subgroup is longer; however, overall, there were no significant differences in the long-term complications in this study.

On the other hand, the consistent application of an anastomosis technique in all patients in a certain period of time prevents selection bias.

Further, a homogeneous patient population, a close follow-up, and a post-treatment schedule, as well as the fact that the interventions were performed by the same surgeon, enable a good comparability.

In conclusion, both the circular stapler and the linear stapler techniques for gastrojejunal anastomosis in laparoscopic Roux-Y gastric bypass are safe techniques with comparable postoperative outcomes and an acceptable rate of complications overall. There were three significant differences: a shorter operation time in favour of LSA, an increased rate of wound infections in CSA, and an increased rate of gastric ulcers as a long-term complication in LSA. The reason for the increased rate of gastric ulcers is not clear, and this may be subject to further observation with a larger patient population. Wound infections in obese patients can be challenging to treat and should be avoided whenever possible. This circumstance in this highly elective procedure requires increased attention.

## Figures and Tables

**Table 1:** Summary of indications and contraindications for bariatric surgery according to the SMOB (Swiss Society for the Study of Morbid Obesity and Metabolic Disorders) criteria.

Indications	Contraindications
BMI ≥35 kg/m^2^	Current pregnancy

Two-year unsuccessful adequate conservative therapy for weight reduction, respectively, one year at BMI ≥50 kg/m^2^	Deep vein thrombosis or pulmonary embolism in the last 6 months
	Unstable angina pectoris
	Cirrhosis of the liver Child B/C
	Pronounced renal insufficiency creatinine ≥300 *μ*mol/l, GFR < 30 ml/min
	Continued substance abuse
	Severe psychological illness not attributable to overweight
	Lack of compliance, unwillingness to participate in postoperative aftercare

**Table 2:** Patient demographics.

Patient demographics	CSA (*n* = 128, 60%)	LSA (*n* = 83, 40%)	*p* value
Age (median–interquartile range)	41 (30–49)	41 (35–48)	0.7334
Female sex (*n* (%))	101 (79)	63 (76)	0.7318
Median BMI preoperative (median–IQR)	42.7 (39.0–47.3)	41.8 (38.3–45.8)	0.4178
Median weight preoperative (median–IQR)	117 (104–132.5)	115 (103–135)	0.6845
Nicotine (*n* (%))	45 (35.2)	31 (37.3)	0.8592
Diabetes (*n* (%))	18 (14.1)	14 (16.9)	0.56

**Table 3:** Surgery/outcome.

Parameter	CSA (*n* = 128, 60%)	LSA (*n* = 83, 40%)	*p* value
Surgery/outcome			
Operation time (median–IQR)	141 (120–178.5)	119 (100–162)	<0.00001
Conversion to laparotomy (*n* (%))	4 (3.1)	0 (0)	0.1558
Weight difference (kg) 1-year FU (median–IQR)	37 (29.2–46.7)	35 (27, 2–40)	0.0576
BMI (kg/m^2^) 1-year FU (median–IQR)	28.6 (25.6–31.7)	29.7 (26, 6–32.9)	0.2177
FU (months) total (median (min–max))	47.0 (12.0–76.4)	14.9 (12, 0–63.1)	<0.00001

FU: follow-up.

**Table 4:** Complications.

Parameter	CSA (*n* = 128, 60%)	LSA (*n* = 83, 40%)	*p* value
Complications			
Early complications (*n* (%))	20 (15.6)	2 (2.4)	0.0022
Anastomosis insufficiency	2 (1.6)	0 (0)	0.2525
Pulmonary embolism	4 (3.1)	0 (0)	0.1039
Wound infection	17 (13.3)	2 (2.4)	0.0143
Late complications (*n* (%))	15 (11.7)	14 (16.9)	0.3918
Gastric ulcer	4 (3.1)	10 (12.0)	0.0238
Internal hernia	12 (9.4)	4 (4.8)	0.3396

## Data Availability

The data used to support the findings of this study are available from the corresponding author upon request.
